# A survey of ivermectin resistance in *Parascaris* species infected foals in south-eastern Poland

**DOI:** 10.1186/s13028-020-00526-2

**Published:** 2020-06-05

**Authors:** Maria Bernadeta Studzińska, Guillaume Sallé, Monika Roczeń-Karczmarz, Klaudiusz Szczepaniak, Marta Demkowska-Kutrzepa, Krzysztof Tomczuk

**Affiliations:** 1 Department of Parasitology and Invasive Diseases, University of Life Sciences in Lublin, 12 Akademicka, 20-033 Lublin, Poland; 2 INRAE/U. de Tours, UMR1282 ISP, 37380 Nouzilly, France

**Keywords:** Anthelmintic resistance, Horse, Ivermectin, Nematode, *Parascaris* spp.

## Abstract

*Parascaris* spp. are major gastro-intestinal nematodes that infect foals and can lead to respiratory symptoms, poor growth, and in some cases obstruction of the small intestine and death. Ivermectin resistance has been reported for *Parascaris* spp. in many countries. In Poland, the knowledge of the level of resistance against ivermectin in *Parascaris* spp. is limited. The aim of this study was to examine the efficacy of ivermectin against *Parascaris* spp. in foals from south-eastern Poland. Foals (n = 225 = reared in 7 stud farms) were treated orally with ivermectin paste. Faecal samples were collected from the rectum of each foal or from the environment straight after defaecation on 1 day prior and 2 weeks after deworming. A faecal egg count (FEC) was performed using the McMaster method with a minimum detection limit of 50 eggs/g. FEC reduction (FECR) was calculated using the Faecal Egg Count Reduction Test. The statistical analysis was limited to foals excreting more than 150 eggs/g before treatment and to stud farms with at least 6 foals excreting at or above this level. Confidence intervals were determined by 1000 bootstraps at farm level and the contribution of sex and age to FECR was quantified using a generalized equation estimation procedure. *Parascaris* spp. eggs were found in 40% of the foals. Following ivermectin treatment, *Parascaris* spp. eggs were identified in 28.4% of the foals. The mean estimated FECR ranged from 44% to 97% and average efficacy was 49.3%. FECR was more pronounced in older foals (P-values = 0. 003). The FECR was more pronounced in males than in females (*P* value = 0.028). This study is the first to indicate a reduced efficacy of ivermectin against *Parascaris* spp. in foals in Poland.

## Findings

*Parascaris* spp. are a major threat for young horses [1]. This infection can lead to respiratory symptoms, poor growth, ill thrift accompanied by rough hair coat and bouts of diarrhoea or colic. In some cases, ascarids can lead to an obstruction and rupture of the small intestine or death of foals [2–4]. In Poland, ivermectin is the most widely used anthelmintic in equine parasite treatment [5, 6]. Drug resistance has been reported for *Parascaris* spp. in other countries [2, 7, 8], but this has not been investigated in Poland.

The aim of this study was to evaluate ivermectin efficacy against *Parascaris* spp. in foals in Poland.

The study was conducted in seven stud farms located in southern or eastern Poland from March to July 2018. The farm details are listed in Table 1. The study included 225 foals of the breeds Arabian, Malopolska, Friesian and Polish half-breed horse of both sexes and aged 3 to 6 months. The stud farms differed in terms of herd size (more than 100 horses, between 50 and 100 and, fewer than 50 horses) (Table 1) and of the management system with pastures available (farms 1, 2, 3 and 5) or not (4, 6, 7) in which case they relied on sandy paddocks with low-growing grass. In all farms, horses were dewormed using orally administered ivermectin paste 3 times a year but for this study, foals were not treated with anthelmintics prior to inclusion. The body weight was estimated visually by experienced stud workers and confirmed according to Rodríguez et al. [9]. The foals were treated orally with ivermectin paste (Paramectin^®^ paste, ScanVet, 0.2 mg per kg of body weight + 10%). The calculated body weight was then increased up to the nearest +50 kg as the scale on the application tube is divided into 50 kg doses. For example, a foal with an estimated body weight of 163 kg was given a dose corresponding to 200 kg (163 + 16.3 = 179.3; rounded up to 200 kg). Deworming was carried out by a qualified veterinary surgeon.

**Table 1:** Stud farm data and prevalence of *Parascaris* spp. egg excretion before and after ivermectin (IVM) treatment

Farm	Herd size	Foals	Sex F/M	Age [3/4/5/6 months]	FEC-positive foals		EPG (min–max)		IVM efficacy^a^
					Pre/with EPG ≥ 150	Post	Pre	Post	Observed FECR
1	≥ 100	49	34/15	6/14/10/19	23/22	20	471 (50–1050)	400 (50–900)	57.0 [36.9–74.2]
2	≥ 100	40	26/14	7/10/9/14	28/24	20	2134 (50–13,750)	1355 (100–5150)	56.8 [20.3–80.3]
3	≥ 100	51	35/16	4/11/17/19	16/15	16	572 (50–1400)	341 (50–2050)	44.0 [6.8–74.5]
4	50–100	24	14/10	4/5/6/9	7/3	2	850 (50–5300)	200 (50-350)	–
5	≤ 50	16	9/7	3/0/7/6	12/8	4	833 (50–5550)	325 (100-650)	86.6 [44.8–96.4]
6	50–100	34	0/34	0/10/6/18	6/4	1	167 (100–250)	50	–
7	≤ 50	11	7/4	1/3/5/2	7/6	1	471 (50–1050)	50	96.9 [92.9–100.0]
Total		225	125/100		99/82	64			

*F* female, *M* male, *FEC* faecal egg count, *EPG* eggs per gram faeces

^a^Observed faecal egg count reduction (Observed FECR) with 95% confidence intervals given in square brackets

Faecal samples were collected from the rectum of each foal or from the environment straight after defaecation. Samples were collected on 1 day prior to and 2 weeks after deworming. Faecal egg counts (FEC) were done by the McMaster method (sucrose-NaCl supersaturated solution with a specific gravity of 1.25) with a minimum detection limit of 50 eggs/g [10].

Faecal egg count (FEC) reduction (FECR) was calculated by the Faecal Egg Count Reduction Test (FECRT) which is a standard method to determine anthelmintic resistance in equine cyathostomin nematodes. Although it has not been validated for *Parascaris* spp., it is currently the only available test for quantifying anthelmintic elimination of reproducing adult female *Parascaris* spp. from individual horses. We followed the guidelines of the American Association of Equine Practitioners [3] providing percent reduction thresholds for diagnosing drug resistance in strongyle populations. In general, a percent efficacy of < 95% for the macrocyclic lactones indicates resistance while a level of 95–98% is interpreted as suspected resistance. These recommendations are primarily made based on equine strongyles, but resistance evaluation for *Parascaris* spp. generally follows the same guidelines [11] and will have to serve until the FECRT has been validated for these.

The statistical analysis was limited to horses with an excretion level of ≥ 150 eggs per gram faeces (EPG) and stud farms with at least 6 horses shedding eggs above this threshold. FECRT 95% confidence intervals were estimated by 1000 bootstraps using the *fecrtCI()* function as implemented in the eggCounts package v2.1–2 [12]. To account for sex- and age-specific variation in *Parascaris* spp. egg excretion dynamics, bootstrapping was performed at the farm level across ages and sexes, and within farm for each age class and sex. The respective effects of these three factors on FECR were also estimated by a marginal modelling approach as previously described [13], using the geeM package v. 0.10.1 [14] assuming a negative binomial distribution for the level of EPG. Under this model, EPG counts are modelled by the sum of environmental effects (sex, age, stud farm), a binary variable coding for the treatment day (accounting for the treatment-associated change in EPG level), and their respective interactions that permits an estimation of the contribution of environmental factors to FECRs.

The detailed data concerning prevalence and EPG are presented in Table 1. *Parascaris* spp. eggs were found in 99 out of the 225 examined foals (40%) with a significant variation among farms ranging from 17.6 to 75%. After deworming, *Parascaris* spp. eggs were identified in 64 foals (28.4%). Ivermectin efficacy was estimated on farms with at least 6 foals with sufficiently high FEC (EPG ≥ 150) (Table 1). Foals from farms 4 and 6 were therefore excluded from the analyses, although a small reduction in FEC was observed in some foals after deworming.

Mean observed FECR values ranged from 44 to 97% efficacy (Table 1) and a mean observed efficacy of 71.1%. Associated confidence intervals did not span the expected 98% efficacy level in four farms, confidence interval ranged from 6.8% to 96.4%. Farm 7 was the only farm showing expected efficacy levels in single foals (> 98%). In this stable, the mean FECR was 97% and the 95% confidence interval ranged from 92.6% to 100%.

**Fig. 1:**
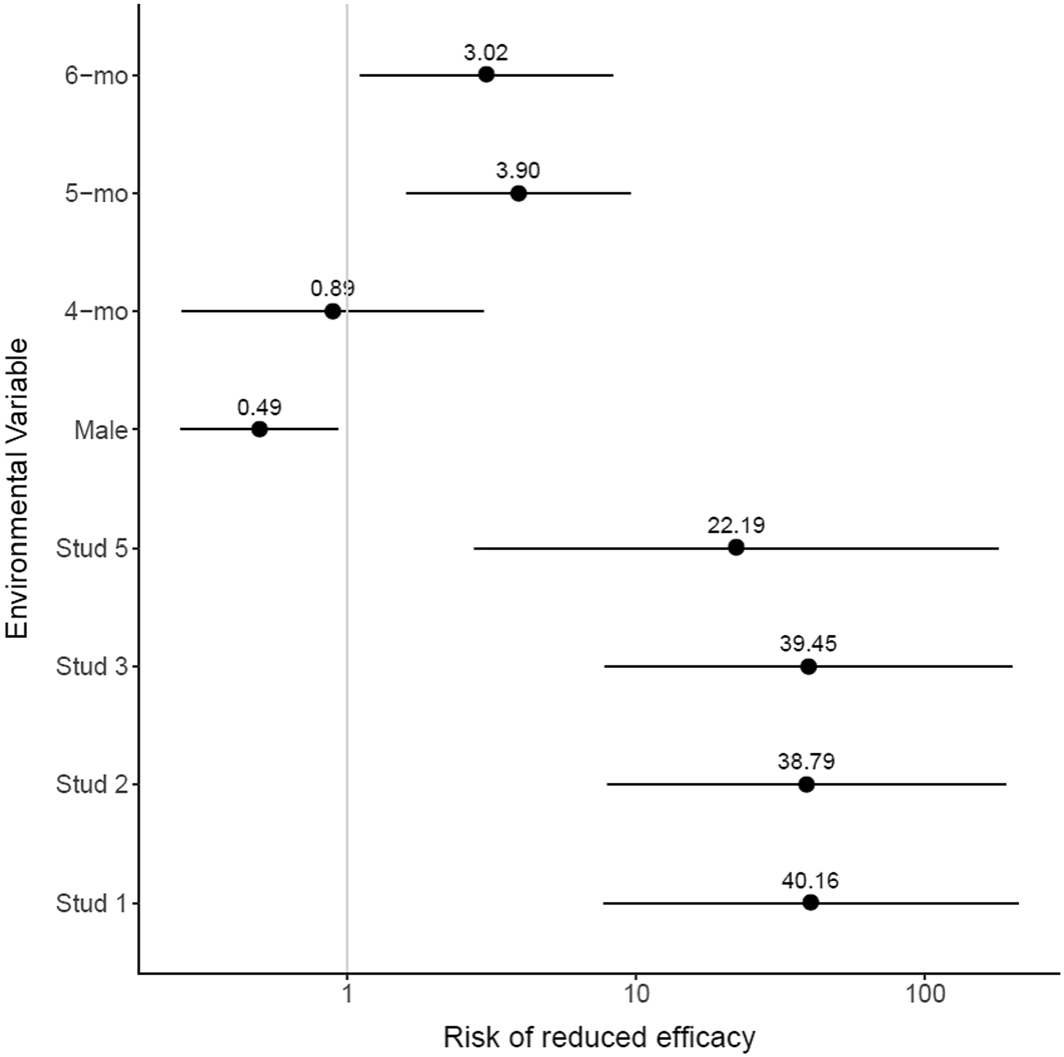
Relative risk of reduced ivermectin efficacy against *Parascaris* spp. was associated with sex, age and farm

Ivermectin was least effective against *Parascaris* spp. infection on farms with the largest herd sizes. Drug efficacy was higher in males compared to females (relative risk of 0.49 [0.27–0.93], P-value = 0.028) (Table 2, Fig. 1), but a reduced efficacy was more common in older foals relative to 3-month-old individuals (relative risks of 3.9 [1.59–9.55] and 3.019 [1.1–8.27] and P-values of 0.003 and 0.03 for 5- and 6-month-old individuals), respectively (Table 2, Fig. 1).

**Table 2:** Estimated mean Faecal Egg Count Reduction following ivermectin treatment with associated confidence intervals by stud farm, sex and age

Study farm		1	2	3	5	7	By variable across stud farms
Foals age (months)	3	42.4 [− 28.63 to 77.0]	93.2 [10.84 to 100.0]	N/A	96.2 [95.495 to 100.0]	N/A	72.2 [35.5 to 94.1]
	4	69.7 [27.6 to 99.6]	82.1 [42.31 to 100.0]	72.9 [50.0 to 100.0]	N/A	N/A	72.3 [38.3 to 95.6]
	5	60.0 [43.0 to 89.5]	35.4 [14.55 to 56.5]	− 18.6 [− 45.349 to 50.0]	85.2 [72.727 to 100.0]	95.6 [90.476 to 100.0]	50.80 [32.7 to 69.0]
	6	61.4 [36.158 to 86.3]	16.3 [0.745 to 39.7]	49.2 [− 20.38 to 88.7]	− 62.5 [− 333.3 to 100.0]	N/A	46.5 [22.2 to 63.5]
Foals sex	Female	50.4 [28.1 to 70.6]	62.9 [14.2 to 87.8]	21.7 [− 35.9 to 63.1]	89.3 [78.6 to 100.0]	95.5 [91.4 to 100.0]	55.8 [36.7 to 71.8]
	Male	75.9 [31.0 to 100.0]	31.8 [14.7 to 58.3]	82.1 [67.3 to 98.3]	84.9 [− 333.3 to 100.0]	N/A	66.7 [40.9 to 81.9]
By farm across age and sex		57.0 [36.68 to 73.1]	56.8 [19.61 to 79.0]	44.0 [4.51 to 75.3]	86.6 [45.79 to 96.6]	96.9 [93.02 to 100.0]

Drug resistance in equine helminths has been reported across a wide range of climatic conditions and management systems [1, 5, 6, 15, 16], and limited efficacy of ivermectin against *Parascaris* spp. has already been described in other countries than Poland [1, 2, 8, 17–19]. This study shows for the first time that ivermectin resistance of *Parascaris* spp. also occurs in Poland.

The difficulty in assessing the efficacy of ivermectin is due to age of animals, their physiological behavior (coprophagia), and the biological properties of *Parascaris* spp. such as female fertility and resistance of eggs to environmental breakdown.

During coprophagia, foals are exposed to “eggs recycling” that can lead to 5% false-positive faecal egg counts in foals [5, 20]. The prepatent period of *Parascaris* spp. ranges from 70 to 110 days. Therefore, it is possible that some of the 3-month-old foals in our study were false-positives, which would explain why FECR was higher in this age group in relation to older foals. However, *Parascaris* spp. egg shedding usually plateaus between 4 and 5 months of age before drastic reduction [21]. This could bias FECR estimates upward in this age group, but this was not the case in our study, thereby supporting true resistance cases. Morris et al. [22] underscore the importance of a control group because *Parascaris* spp. FEC may be either naturally increasing or declining over the 14-day period used to conduct the FECRT. In our study, though, we were not able to create a control group as there was no possibility of isolating the foals. The main reason for this was reluctance among the owners and managers to leave the groups untreated.

The risk factors underpinning drug resistance in equine helminths are poorly characterized and the sole available estimates were quantified in strongyle populations [23, 24]. Hence it is unclear what factors are critical in the development of drug resistance in *Parascaris* spp. populations. Of note, *Parascaris* spp. populations sampled across northern Europe, North and South-America exhibited limited genetic diversity [25]. This forms a limit to maintain and select allelic variants conferring resistance to anthelmintic drugs. However, reducing the prevalence of infection would help reduce drug usage and ultimately their efficacies. Hautala et al. [16] found that *Parascaris* spp. infection was heavily affected by farm size and the frequency of horse movements. Horses from large breeding farms were more likely to shed *Parascaris* spp. eggs as found in our study. Maintaining a better environmental hygiene, e.g. faeces removal, could also contribute to a decrease in the prevalence of infections for that species [26, 27].

The genetic basis underpinning ivermectin resistance is yet to be identified. Recent work has demonstrated that the transmembrane efflux pumpP-glycoprotein-11 was associated with ivermectin resistance in *Parascaris* spp. [28]. This may serve as a tool for monitoring drug resistance in the field.

To limit the prevalence of drug resistance, in silico studies [29] suggested that first anthelmintic treatment should be administered only twice at the age of 2 and 5 months to allow foals to develop a sufficient immune response to *Parascaris* spp. while leaving enough refugia to the worm population. However, these latter results are based on simulations that have not been validated in the field to date.

The use of herbal preparations may represent a useful alternative for the management of *Parascaris* spp. infection. For example, extract of *Artemisia dracunculus*, *Mentha pulegium*, *Zataria multiflora* have potential to be used as anthelmintic for the control of ascariasis in horses [30]. This requires further research on their activity. *Parascaris* spp. larval culture is possible [31] but in vitro screening assays still need to be developed and validated for this species.

## Data Availability

The datasets used and/or analysed during the current study are available from the corresponding author on reasonable request.

## Abbreviations

FEC: Faecal egg count
FECR: Faecal egg count reduction
FECRT: Faecal egg count reduction test
